# A Survey of Physicians’ Knowledge and Practices Towards Oral Appliance Therapy for Obstructive Sleep Apnea Treatment

**DOI:** 10.3390/dj13020063

**Published:** 2025-01-30

**Authors:** Chaniporn Ananwattananon, Supatchai Boonpratham, Yodhathai Satravaha, Chaiyapol Chaweewannakorn, Supakit Peanchitlertkajorn

**Affiliations:** Department of Orthodontics, Faculty of Dentistry, Mahidol University, Bangkok 10400, Thailand; chaniporn.ana@mahidol.ac.th (C.A.); supatchai.boo@mahidol.ac.th (S.B.); yodhathai.sat@mahidol.ac.th (Y.S.); chaiyapol.cha@mahidol.ac.th (C.C.)

**Keywords:** dental sleep medicine, medical education, obstructive sleep apnea, oral appliance therapy, physician knowledge, orthodontics

## Abstract

Background: Oral appliance therapy (OAT) is an effective treatment alternative for obstructive sleep apnea (OSA). Understanding physicians’ knowledge and practices regarding OAT is crucial for improving OSA treatment, particularly in Southeast Asia, where there is limited research on this topic. Objectives: This study sought to survey physicians’ knowledge and practices regarding OAT for OSA treatment. Methods: A descriptive cross-sectional survey was conducted among Thai physicians attending the annual Sleep Society of Thailand meeting. The questionnaire included questions on demographics (six items), educational background (four items), OAT knowledge (two items), indications (three items), effectiveness (seven items), referral practices (five items), treatment planning (eight items), and the importance of OAT education (one item). Descriptive and comparative analyses were performed using SPSS to assess differences in knowledge and referral practices across various demographics. Results: This study achieved a response rate of 51.7% (30/58), with the respondents averaging 37.8 ± 6.0 years in age and 13.7 ± 6.7 years in practice. Although over 66.7% of the respondents recognized OAT’s effectiveness and understood its indications, only 36.7% regularly prescribed OAT, and 57.7% referred patients to specific dentists. Knowledge and referral practices differed significantly based on years of practice and postgraduate training in sleep medicine. Physicians with 11–20 years of practice demonstrated higher pediatric OAT knowledge scores compared to those with less experience. Those with postgraduate training achieved higher adult OAT knowledge scores and had greater referral experience. Conclusions: Despite the general awareness and good knowledge of OAT among physicians, clinical use and referral rates remain low. To bridge the gap between knowledge and practice, it is recommended that postgraduate training in dental sleep medicine is promoted and further research is conducted to identify barriers to the adoption of OAT.

## 1. Introduction

Obstructive sleep apnea (OSA) is an increasingly common disorder, with a prevalence of 13–33% in men and 6–19% in women [1]. People of all ages can be affected by OSA. Adults aged 65 years and older were reported to have the highest prevalence, ranging from 28% to 43% [2]. Pediatric obstructive sleep apnea (POSA) has a prevalence of 2–3%, with a peak incidence observed between 2 and 8 years of age [3]. OSA is characterized by the repetitive collapse of the upper airway during sleep, causing intermittent hypoxia, intrathoracic pressure swings, sympathetic surges, and sleep fragmentation [4]. Patients with OSA have increased risks of motor vehicle accidents [5], hypertension, cardiovascular diseases, stroke [6,7], and poor quality of life [8]. Children who are left untreated could develop neurocognitive deficits, impaired executive function and learning, behavioral problems, poor attention span, hyperactivity [9,10], cardiovascular sequelae, and metabolic disorders [11].

The management of individuals diagnosed with OSA necessitates a multidisciplinary approach. There are many surgical and non-surgical treatment options available [12]. Continuous positive airway pressure (CPAP) is currently regarded as the gold standard treatment for adult OSA across all degrees of severity. It is also the most commonly used treatment modality [12]. CPAP has demonstrated a high level of success in decreasing respiratory events. However, past studies demonstrate that patient’s acceptance, tolerance, and adherence to CPAP are suboptimal, hence diminishing its overall efficacy [13,14]. In contrast, oral appliance therapy (OAT) has emerged as a popular non-surgical treatment method [15]. Previous investigations showed that patients using OAT reported higher levels of compliance and adherence compared to those using CPAP therapy [16,17]. Although CPAP is more efficacious in reducing the apnea–hypopnea index (AHI), the overall effectiveness of OAT is comparable due to its superior adherence among patients [18]. There is a considerable amount of research in the literature demonstrating the efficacy of OAT in alleviating OSA and improving associated health outcomes [19]. According to a joint clinical practice guideline issued by the American Academy of Sleep Medicine and the American Academy of Dental Sleep Medicine [20], OAT is recommended as the primary treatment for patients with primary snoring as well as those with mild-to-moderate OSA. It is also recommended for patients with severe OSA who fail treatment with CPAP [20]. Generally, oral appliances can be categorized into three main types: soft palate lifters, tongue-retaining devices, and mandibular advancement devices (MADs) [21]. Among these, custom-made titratable MADs are the recommended type of oral appliance for the treatment of OSA [20]. These devices should be designed to position the mandible in a protruded position and allow for gradual advancement (titratable) to prevent upper airway collapse during sleep and achieve an optimal respiratory response [20].

Physicians play a vital role in diagnosing and treating OSA, determining treatment based on the severity of the condition, the presenting symptoms, and the underlying causes of upper airway obstruction. Additionally, they should consider patient preferences, adherence potential, and lifestyle when formulating a treatment plan [22]. It is essential to present and discuss all treatment options as well as their benefits and risks with patients [23,24]. Some patients may require treatment by various specialists and should be managed by a multidisciplinary sleep team [25]. A multidisciplinary approach is essential for effectively managing OSA by enabling specialized care from various healthcare professionals. This collaboration enhances communication and enables personalized treatment strategies that improve patient adherence and health outcomes by addressing each patient’s unique needs, ensuring effective treatment for OSA [26].

Physicians’ knowledge and attitudes regarding a particular treatment can impact their decision to provide patient care and treatment [23]. Past studies highlight the significant gaps in physicians’ knowledge and perceptions regarding the management of OSA [27,28,29]. Jauhar et al. (2008) identified significant variability in the management of OSA by medical practitioners in Scotland, highlighting inconsistencies in screening practice and a lack of standardized protocols for OAT. As a result, some patients received comprehensive care, while others experienced inconsistent screening and treatment. This led to disparities in the quality of OSA management [27]. The study conducted by Meenakshi et al. (2017) revealed that 98% of medical practitioners in India were unaware of OAT for the management of OSA [29]. Similarly, a study by Alrejaye et al. (2022) in Saudi Arabia indicated that physicians lacked sufficient knowledge about OSA and its management, including OAT [28]. Overall, these findings demonstrate that the level of OAT knowledge among physicians remains consistently low over time and across different parts of the world.

The Southeast Asian context presents distinct challenges and opportunities in the management of OSA. Previous studies indicated that Asian populations experience greater severity of OSA than Caucasians, even in the absence of obesity, suggesting that distinct anatomical and physiological factors are at play [30,31]. Additionally, cultural beliefs, socioeconomic factors, healthcare accessibility, and varying levels of awareness of sleep disorders significantly influence the perception and treatment of OSA in this region [32]. Although studies on this topic have been conducted in Europe and the Middle East, their findings may not be entirely applicable to East and Southeast Asian countries due to differences in race and ethnicity, geography, culture, and socioeconomic status. Notably, there is a lack of published studies on this topic conducted in Southeast Asia. Understanding physicians’ knowledge and perspectives on OAT is crucial for improving treatment. Therefore, this study aimed to fill this research gap by surveying Thai physicians’ knowledge and practices regarding OAT for OSA treatment.

## 2. Materials and Methods

### 2.1. Research Participants

All physicians who attended the annual meeting of the Sleep Society of Thailand in November 2022 were invited to participate in this study. The research objectives were explained to them verbally. The physicians also received written information describing the study objectives and questionnaire details, along with a QR code that directed them to the questionnaire. They were guaranteed that their responses would be both anonymous and untraceable. They were given 30 days to complete the survey. This study was approved by the Ethics Committee of Human Research, Faculty of Dentistry and Faculty of Pharmacy, Mahidol University, Bangkok, Thailand (no. MU-DT/PY-IRB 2022/003.1701).

### 2.2. Questionnaire Construction

A questionnaire in English was developed for the purpose of conducting this descriptive cross-sectional survey. The survey questions were designed in consultation with a sleep medicine specialist, a dental sleep medicine specialist, an orthodontist, and a statistician. The questionnaire’s validity was confirmed by three experts (two sleep medicine specialists and one dental sleep medicine specialist) using the index of item objective congruence. The questionnaire underwent iterative revisions until the objective congruence for each item exceeded 0.5 [33]. The pilot questionnaire was evaluated by 20 physicians to assess the test–retest reliability. An intraclass correlation coefficient of 0.8 was obtained, indicating good reliability [34]. The final and electronic version of the questionnaire was created using Google Forms (Google Inc., Mountain View, CA, USA).

The questionnaire consisted of eight sections, with a total of 36 questions (Supplementary Material S1).

Part 1 (demographic data) comprised six questions collecting the respondent’s demographic data, including age, sex, years in practice, professional status, specialty, and type of practice.

Part 2 (basic medical education and postgraduate training) included four questions exploring the educational background and training experiences of the respondents regarding OAT. The questions assessed their formal education on OAT that was received during their basic medical education and postgraduate training.

Part 3 (OAT knowledge) included two multiple-choice questions that investigated the respondent’s knowledge of oral appliances used for the treatment of OSA in both adult and pediatric patients. In this part, both the “adult OAT knowledge score” and the “pediatric OAT knowledge score” were determined by awarding one point for each correct answer, with a maximum score of two points for each category.

Part 4 (indication) assessed knowledge regarding the indications and usage of OAT in individuals with varying degrees of OSA severity. It included three questions answered on a five-point Likert scale, allowing the respondents to express their level of agreement with statements regarding OAT recommendations. One point was awarded for each question when the respondents answered “agree” or “strongly agree”. The total score for this part was tallied as the “indication score”, with a maximum of three points.

Part 5 (effectiveness) evaluated the perceived effectiveness of OAT using seven items. The respondents used a five-point Likert scale to indicate their level of agreement with those seven items. One point was awarded for each question when the respondents answered “agree” or “strongly agree”. This scoring method captured varying degrees of perceived effectiveness among the respondents. The total score for this part was calculated as the “effectiveness score”, with a maximum of seven points.

Part 6 (referral practice) was composed of five questions investigating the referral practice of the respondents regarding OAT. It included questions on whether the respondents had ever referred patients for these treatments (referral experience), reasons for not referring them, the frequency of referrals in the past 12 months, and whether they had a regular dentist to whom they referred patients (referral dentist).

Part 7 (treatment planning) consisted of eight multiple-choice questions designed to explore the decision-making processes and treatment planning strategies of the respondents regarding OAT for OSA. This part investigated the treatment options that the respondents recommended based on the severity of OSA, the criteria they used for referring patients’ need for OAT, and specific circumstances in which they considered OAT a first-line treatment. Additionally, these questions examined the type of information the respondents typically provided to patients before making a referral for OAT and identified the key specialists involved in a multidisciplinary team approach to managing OSA.

Part 8 included a single open-ended question aimed at understanding the respondents’ perceptions of the importance of education in OAT for the treatment of OSA. Specifically, it asked whether the respondents believed that physicians should be educated about these treatment options and invited them to provide reasons for their answers.

### 2.3. Statistical Analyses

Statistical analyses were conducted using SPSS for Windows (version 22.0; IBM Corp., Armonk, NY, USA). Each completed survey was assigned a unique identification number, and all data were imported into Microsoft Excel v15.3 (Microsoft, Redmond, WA, USA) for initial processing. Descriptive analysis was applied to several key areas of the study, including demographic characteristics, such as age, years of practice, professional status, specialty, and workplace; educational and training experiences related to oral appliances for OSA; knowledge and familiarity with different types of oral appliances; perceptions of treatment and its effectiveness; the practice of referrals, including frequency, patterns, and reasons for referral; and decision-making and treatment planning, covering recommended treatment options, criteria for referrals, conditions under which OAT was considered, and the composition of the multidisciplinary team involved in managing OSA. All these findings were reported as frequencies and percentages.

For further analysis, data were categorized based on four demographic variables: age (21–40, 41–60 years), years in practice (0–10, 11–20, >20 years), postgraduate training in sleep medicine (trained vs. not trained), and whether respondents received education on oral appliances for OSA during their basic medical education (educated vs. not educated). These groups were analyzed across four domains: the adult OAT knowledge score, the pediatric OAT knowledge score, the indication score, and the effectiveness score.

The data distribution was assessed using the Shapiro–Wilk test. Continuous variables were summarized using medians and interquartile ranges (IQRs), whereas categorical variables were presented as frequencies and percentages. Non-parametric statistical tests were performed to analyze the differences between groups. The Mann–Whitney U test was used to compare the two groups, whereas the Kruskal–Wallis test was employed for comparisons among three or more groups. Post hoc pairwise comparisons were conducted using Dunn’s Bonferroni adjustment when significant differences were detected.

Referral practice was further examined across two domains: referral experience and referral to a dentist. Fisher’s exact test was used to analyze significant differences in referral practice based on the same demographic variables previously mentioned. The variable “referral experience” was used to categorize respondents who had previously referred patients for OAT and those who had not. “Referral dentist” was assessed by determining whether the respondents had a dentist to whom they could refer patients for OAT. A *p*-value of 0.05 was considered statistically significant for all analyses.

## 3. Results

### 3.1. Demographic Data and Educational Experience

This study achieved a final response rate of 51.7% (30/58). Among the respondents, 73.3% (n = 22) were female, with an average age of 37.8 years (SD = 6.0). The respondents had 13.7 years (SD = 6.7) of practice on average. The majority (53.3%) of the participants were specialists, including sleep medicine specialists, otolaryngologists, and neurologists. Regarding education and training in sleep medicine, 76.7% had received postgraduate training in sleep medicine. Eighty percent worked in medical schools or government hospitals, whereas the remainder were in private practice. The demographic characteristics of the respondents are shown in Table 1. Key sources of information on OSA and OAT included academic conferences (66.7%), postgraduate medical education (56.7%), and medical journals (36.7%).

### 3.2. OAT Knowledge

Eighty percent of the respondents considered MADs as a treatment option for adult patients with OSA, while only 43.3% regarded a tongue-retaining device as an option. These responses were calculated to obtain the adult OAT knowledge score, yielding a median score of 1.5 (IQR = 0.75–2.0). For POSA, 70% considered rapid maxillary expansion as a treatment option, whereas only 20% regarded functional appliance as an option. These responses were used to calculate the pediatric OAT knowledge score, resulting in a median score of 1.0 (IQR = 0–1.0).

### 3.3. Indications for OAT

Seventy percent agreed that OAT could be recommended as a first-line treatment for adult patients with primary snoring. Additionally, 76.7% of the respondents agreed that OAT is suitable as a first-line treatment for adult patients with mild-to-moderate OSA. For adult patients with severe OSA who failed CPAP therapy, 66.7% of the respondents suggested OAT as a recommended treatment option. These responses were calculated to obtain an indication score with a median score of 2.0 (IQR = 1.0–3.0).

### 3.4. Perceived Effectiveness of OAT

**Figure 1:**
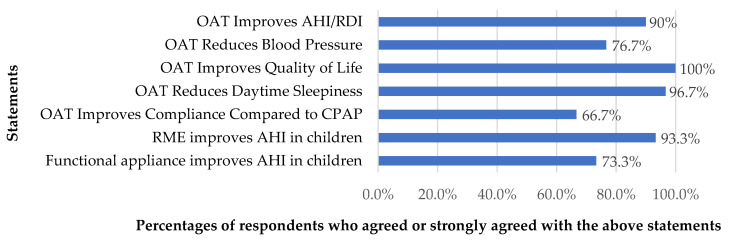
The percentages of respondents who agreed or strongly agreed with statements regarding the effectiveness of OAT for OSA. AHI, apnea–hypopnea index; CPAP, continuous positive airway pressure; OAT, oral appliance therapy; RDI, respiratory disturbance index; RME, rapid maxillary expansion.

The results also showed that most respondents agreed on the effectiveness of OAT, affirming that OAT enhanced various aspects of OSA management in adults. These improvements included the AHI, respiratory disturbance index (RDI), oxygen saturation, blood pressure, quality of life, daytime sleepiness, and compliance of patients. Regarding the effectiveness of OAT in POSA, most respondents also agreed that rapid maxillary expansion and functional appliances were effective at reducing AHI and OSA severity. These responses were calculated to obtain the effectiveness score, yielding a median score of 6.0 (IQR = 5.0–7.0). The percentages of respondents who agreed and strongly agreed with the statements regarding the effectiveness of OAT for OSA are presented in Figure 1.

### 3.5. Referral Practice

Most respondents (86.7%) referred patients for OAT at least once. More than half (57.7%) also reported that they had dentists who regularly referred patients for OAT. However, 13.3% never referred patients due to reasons such as using other treatment modalities, the lack of available dentists, and insufficient knowledge about OAT. Regarding the referral frequency, 57.7% of the respondents made fewer than five referrals in the past 12 months. In total, 34.6% referred 5 to 10 adult patients for OAT over the same period. In contrast, referrals for POSA were lower, with 65.4% of the respondents not making any referrals at all, 23.1% making fewer than five referrals, and only 11.5% making 5 to 10 referrals over the past 12 months.

### 3.6. Comparison of Physicians’ Knowledge Scores Across Different Demographic Groups

This study analyzed four key knowledge scores: the adult OAT knowledge score, the pediatric OAT knowledge score, the indication score, and the effectiveness score. The objective was to assess significant differences in these scores based on demographic variables, including age, years in practice, postgraduate training in sleep medicine, and basic medical education.

When examining the years of practice across the three groups (0–10 years, 11–20 years, and over 20 years), a significant difference was identified in the pediatric OAT knowledge score (*p*-value = 0.049). Specifically, physicians with 11–20 years of practice demonstrated a significantly higher pediatric OAT knowledge score compared to those with fewer than 10 years of practice (*p*-value = 0.045). Moreover, the analysis demonstrated that physicians who had completed postgraduate training in sleep medicine scored significantly higher on the adult OAT knowledge score (*p*-value = 0.004) compared to those without such training. On the contrary, no significant differences were observed in any of the knowledge scores when comparing the respondents based on age (21–40 years vs. 41–60 years) or basic medical education (educated vs. not educated) (*p*-value > 0.05). The detailed findings are presented in Table 2.

### 3.7. Comparison of Physicians’ Referral Practices Across Different Demographic Variables

Regarding referral experience, a significant difference was observed between those who had postgraduate training in sleep medicine and those who did not (*p*-value = 0.031). Specifically, 95.7% of the respondents with previous training reported having referral experience at least once, compared to only 57.1% of those without this training. In contrast, no significant differences in referral experience were found when comparing age groups (21–40 years vs. 41–60 years), years in practice (0–10, 11–20, and over 20 years), or basic medical education (educated vs. not educated).

In terms of referral dentists, a significant difference based on the number of years in practice was identified (*p*-value = 0.047). Specifically, 44.4% of practitioners with 0–10 years of experience, 37.5% of those with 11–20 years, and 100% of those with over 20 years in practice reported that they had a dentist to whom they regularly referred patients for treatment. These findings indicate that practitioners with more than 20 years of experience were significantly more likely to refer patients compared to those with fewer years in practice. No significant differences were observed when comparing the respondents based on age groups (21–40 years vs. 41–60 years), postgraduate training in sleep medicine (trained vs. not trained), or basic medical education (educated vs. not educated). The detailed findings are presented in Table 3.

### 3.8. Factors Influencing the Decision to Use OAT

Approximately one-third of the respondents (36.7%) recommended OAT as a treatment option for adult patients with OSA, while CPAP was recommended by most at 96.7%. The key factor influencing the decision to refer patients to OAT was CPAP intolerance (83.3%), followed by patient preference (70%) and the severity of OSA (43.3%).

When focusing on the severity of OSA, OAT was mostly considered for patients with milder conditions (66.7% for mild OSA and 53.3% for primary snoring). As the severity increased, the recommendation for OAT significantly decreased. Only 40% of the respondents considered OAT for patients with moderate OSA, and a mere 13.3% recommended it for those with severe OSA. As for recommending OAT as a first-line treatment, the most common diagnosis was primary snoring (46.7%), followed by mild OSA (43.3%). Only 20% considered it for moderate OSA, and a mere 3.3% considered it for severe OSA. Notably, 30% of the respondents stated that they had never recommended OAT as a first-line treatment for adult patients with OSA.

Before referring patients for OAT, the most discussed topics were the advantages of the therapy (63.3%) and the treatment mechanism (56.7%). Disadvantages and effectiveness were each explained by 53.3% of the respondents, while cost was discussed by 46.7%. Fewer respondents mentioned complications (36.7%), the time required for treatment (30%), types of appliances (20%), and insurance coverage (16.7%).

### 3.9. Physicians’ Perspectives on the Role of Dentists in a Multidisciplinary Approach

**Figure 2:**
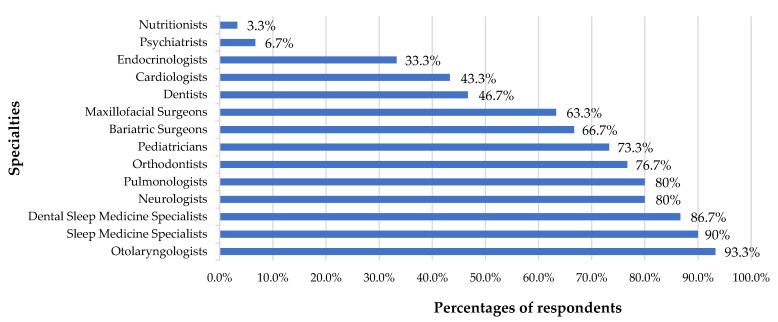
The percentage of respondents recognizing these healthcare specialties as part of a multidisciplinary team for OSA treatment.

The questionnaire revealed that several medical and dental specialists were recognized as part of the multidisciplinary team for OSA treatment. Specifically, 86.7% of the respondents acknowledged the role of dental sleep medicine specialists, and 76.7% acknowledged the role of orthodontists. For further details, see Figure 2.

### 3.10. Importance of Educating Physicians on OAT for OSA Treatment

Most respondents (86.7%) strongly supported the notion that physicians should receive education in OAT for managing OSA. They highlighted several reasons for their support, including a lack of knowledge regarding OAT, a lack of OAT education in their basic medical education, the importance of understanding OAT to comply with current standards of care, and the ability to offer patients a wider array of treatment options. Moreover, most respondents also believed that it is essential to be well informed about all available treatment options to ensure appropriate referrals and comprehensive care.

## 4. Discussion

Our study found that approximately three-quarters of respondents (76.7%) received formal training in sleep medicine, indicating significant clinical experience in sleep medicine. Most respondents demonstrated knowledge consistent with guidelines for selecting and prescribing OAT for patients with varying degrees of OSA severity and patients who failed CPAP therapy [20]. Furthermore, they were knowledgeable of the effectiveness of OAT, noting its positive impact on AHI/RDI, blood pressure, quality of life, and daytime sleepiness, with a higher compliance rate compared to CPAP. These findings collectively indicate that most respondents possess a good understanding of OAT. Results from other studies revealed that awareness levels regarding OAT for managing OSA varied significantly across regions. For example, approximately 98% of medical practitioners in India lacked awareness of OAT, as reported by Meenakshi et al. (2016) [29]. In Saudi Arabia, about 31% of physicians were not familiar with OAT, according to Alrejaye et al. (2022) [28]. In our study, approximately 20% of respondents did not recognize MADs as a treatment option for OSA patients. This study also revealed that 86.7% of respondents referred patients for OAT at least once. However, it is important to note that this reflected the minimal threshold of a single referral, which does not necessarily indicate substantial experience. Additionally, only 57.7% of these physicians had specific dentists for regular referrals. In contrast, Meenakshi et al. (2016) reported a mere 1.2% referral rate from medical practitioners to orthodontists in India [29]. However, it appears that more recent studies demonstrate higher levels of awareness compared to older ones. Referral rates also increased as knowledge improved. The detailed findings from previous studies are presented in Table 4.

Despite the overall good level of knowledge on the effectiveness of OAT, only 36.7% of the respondents regularly recommended OAT compared to 96.7% for CPAP in adult patients with OSA. The gap between knowledge and actual recommendations could be due to several factors. First, CPAP has long been established as the gold standard treatment, and physicians are more familiar with it. It offers superior polysomnographic outcomes and requires fewer appointments to initiate. Conversely, although OAT has only gained popularity in the past decade, it necessitates more visits and interdisciplinary collaboration. OAT is not suitable for all patients. It is not recommended for edentulous patients and those with an insufficient number of teeth, limited mandibular movement, or periodontal disease [35]. Petit et al. (2002) estimated that up to 34% of OSA patients are ineligible for OAT due to these dental limitations [36]. OAT is more effective in patients with impaired upper airway anatomy and less effective for individuals with non-anatomical phenotypes, such as impaired muscle function or a low respiratory arousal threshold [37]. Therefore, considering the patient’s phenotype is essential when determining the suitability and potential effectiveness of OAT.

The lack of qualified dentists for OAT significantly restricted its access, given the absence of dental sleep medicine training in both postgraduate and basic dental education programs. A recent study by Peanchitlertkajorn et al. (2024) highlighted disparities and inadequacies in dental sleep medicine education within postgraduate orthodontic programs in Thailand [38]. Similarly, the findings reported by Sangalli et al. (2024) indicated significant disparities in the availability of dental sleep medicine courses across U.S. postgraduate dental programs. Among the 68 programs surveyed, only 7.5% of faculty members received formal training in dental sleep medicine, and only 2.6% were board-certified in this specialty. Furthermore, only 41.8% of these programs offered any form of dental sleep medicine education [39]. These findings highlight the urgent need for enhanced dental sleep medicine education to address the growing involvement of dental professionals in managing sleep-related disorders, as the current shortage of specialists may result in missed opportunities for comprehensive OSA management for patients [38,39].

Finally, insurance coverage also significantly impacts the utilization of OAT. In Thailand, only CPAP is covered by both public and private health insurance, but OAT, despite its lower cost, is not covered. Research conducted by Liptak et al. (2021) indicated that CPAP is 3.9 times more expensive per day compared to OAT. Initially, CPAP may appear to be the more economical option. However, over the long term, it incurs ongoing costs associated with equipment replacement and maintenance. In contrast, OAT generally involves fewer recurring costs following the initial setup, which results in CPAP being more expensive in the long term [40]. Reducing the financial barriers associated with OAT by expanding insurance coverage and integrating it into public health policy could enhance patient access. For instance, Norway includes OAT in public health plans to facilitate collaboration within multidisciplinary centers [41]. This integrated approach not only enhances treatment quality but also streamlines referrals and promotes interdisciplinary collaboration.

This study also found that clinical experience (measured by the number of years in practice) and training in sleep medicine had positive effects on knowledge of OAT and referral experience. Our findings demonstrate that physicians with postgraduate training in sleep medicine are more knowledgeable about OAT and have more experience referring patients for OAT compared to those without training (95.7% vs. 57.1%). These findings are consistent with those reported by Alrejaye et al. (2022), highlighting the critical roles that clinical experience and specialty training play in enhancing physicians’ understanding and prescribing of OAT [28]. These findings underscore the importance of integrating comprehensive sleep medicine training, including OAT education, into physician training to improve the quality of care for patients with sleep disorders.

The referral rate for pediatric patients with OSA is significantly lower than that for adults. Our study demonstrated that physicians had less knowledge of oral appliances for children compared to adults, with only about 10% of physicians recommending OAT for mild OSA and 30% for moderate-to-severe cases. This lower referral rate may be attributed to the lower prevalence of POSA compared to adults, as well as the absence of established practice guidelines specifically addressing OAT for children. Additionally, there is limited clinical evidence supporting the efficacy of OAT in pediatric patients, which may affect physicians’ willingness to make referrals. Therefore, there is a pressing need for more research and clearer guidelines regarding the use of oral appliances in managing POSA.

Most of the respondents recognized the roles of sleep medicine specialists (90%) and orthodontists (76.7%) as multidisciplinary team members in managing OSA. They also overwhelmingly supported the need for physician education on OAT. Many physicians recognized a lack of OAT knowledge from their basic medical education. This indicates a need to include OAT education at this level. Additionally, OSA treatment requires a multidisciplinary approach involving various types of specialists. Physicians need to be well versed in all available treatment options to make appropriate referrals. Therefore, it is important to promote additional training for physicians to become more knowledgeable and confident in prescribing OAT. Improving interdisciplinary collaboration between medical and dental practitioners is also important to ensure comprehensive patient care.

This study was the first survey conducted in Southeast Asia, providing valuable baseline information on physician knowledge and practices regarding OAT. Although the research was conducted in Thailand, the results are likely applicable to other developing nations in Southeast Asia with similar geographic and socioeconomic characteristics. This study also had some limitations, including a moderate response rate that resulted in a small sample size (n = 30). The moderate response rate could introduce a non-response bias, while the small sample size may not adequately represent the broader physician population, particularly when stratified by demographic groups. Additionally, because this survey was conducted during an annual meeting of the Sleep Society of Thailand, there could be potential selection bias, as participants could have had pre-existing interests or expertise in sleep medicine. Moreover, our study did not investigate the barriers to referrals for all participants. We only focused on those who had never referred patients for OAT. Consequently, this limited our ability to explore the barriers that could impact the majority of respondents and contribute to low referral practices. Nonetheless, the findings from this study offer a valuable basis for understanding OSA management practices in comparable settings, providing a framework for analyzing how similar factors might influence treatment approaches in other regions. Future research should investigate barriers to the adoption of OAT in clinical practice, as well as a more diverse group of physicians, to enhance the robustness and generalizability of the findings. Additionally, this study focused exclusively on physicians, but dentists’ perspectives are also crucial. Dental professionals can play a significant role in recognizing OSA signs and symptoms, making referrals, and treating patients with OSA. Therefore, investigating dentists’ knowledge and practices regarding OSA and OAT would also be very valuable. Furthermore, a comparative study between the knowledge and practice of physicians and dentists can be conducted to understand the factors influencing their perspectives on this issue.

## 5. Conclusions

This study revealed that physicians had adequate knowledge regarding the effectiveness of OAT. However, the clinical usage of OAT remained limited. This study also found that work experience and postgraduate training in sleep medicine had positive effects on both knowledge of OAT and referral practices. To bridge the gap between the knowledge of OAT and referral practices, it is essential to promote postgraduate training in dental sleep medicine for physicians while also encouraging further research to examine the barriers to the adoption of OAT in clinical practice for the effective management of OSA.

## Figures and Tables

**Table 1:** Demographic characteristics of study participants.

Sex	Female	8 (26.7%)
	Male	22 (73.3%)
Age (year)	21–30	1 (3.3%)
	31–40	22 (73.3%)
	41–50	5 (16.7%)
	51–60	2 (6.7%)
Years in practice (year)	0–10	13 (43.3%)
	11–20	11 (36.7%)
	21–30	4 (13.3%)
	31–40	2 (6.7%)
Professional status	Practicing specialist physicians	16 (53.3%)
	Practicing non-specialist physicians	4 (13.3%)
	Residents/fellows	10 (33.3%)
Workplace	Government hospitals and medical schools	24 (80%)
	Private clinics and private hospitals	6 (20%)

**Table 2:** Comparison of the adult OAT knowledge score, the pediatric OAT knowledge score, the indication score, and the effectiveness score across different demographic variables.

	Group	Adult OAT Knowledge Score (Full Score = 2)		Pediatric OAT Knowledge Score (Full Score = 2)		Indication Score (Full Score = 3)		Effectiveness Score (Full Score = 7)	
		Median	*p*-Value	Median	*p*-Value	Median	*p*-Value	Median	*p*-Value
Age	21–40	1 (1–2)	0.873	1 (0–1)	0.288	2 (2–3)	0.174	6 (5–7)	0.666
	41–60	2 (0–2)		1 (1–1)		1 (1–3)		6 (5–7)	
Years in practice	0–10	1.5 (1–2)	0.090	1 (0–1)	0.049 *	3 (2–3)	0.156	7 (5–7)	0.267
	11–20	1 (0–2)		1 (1–2)		2 (1.7–3)		6 (5.7–7)	
	>20	2 (0–2)		1 (0.7–1)		1.5 (1–2.2)		5 (4.2–6.2)	
Basic medical education	Yes	2 (1–2)	0.415	1 (0–1)	0.884	2 (1–3)	0.325	7 (5–7)	0.755
	No	0.5 (0–2)		1 (1–1)		2.5 (2–3)		6 (5.2–7)	
Training in sleep medicine	Yes	2 (1–2)	0.004 *	1 (1–1)	0.174	2 (1–3)	0.631	6 (5–7)	0.666
	No	0 (0–1)		1 (0–1)		2 (1–3)		6 (6–7)	

Note: * denotes a statistically significant difference.

**Table 3:** Comparison of referral experience and referral dentists across different demographic variables.

	Group	Referral Experience			Referral Dentist		
		Yes	No	*p*-Value	Yes	No	*p*-Value
Age	21–40	82.6%	17.4%	0.548	47.4%	52.6%	0.178
	41–60	100%	0%		85.7%	14.3%	
Years in practice	0–10	81.8%	18.2%	0.643	44.4%	55.6%	0.047 *
	11–20	80%	20%		37.5%	62.5%	
	>20	100%	0%		100%	0%	
Basic medical education	Yes	94.4%	5.6%	0.274	52.9%	47.1%	0.683
	No	75%	25%		66.7%	33.3%	
Training in sleep medicine	Yes	95.7%	4.3%	0.031 *	63.6%	36.4%	0.279
	No	57.1%	42.9%		25%	75%	

Note: * denotes a statistically significant difference.

**Table 4:** Overview of findings from previous studies regarding physician knowledge, awareness, and referral practices for OAT in OSA across different regions.

Authors, Year	Study Site	Findings
Jauhar et al., 2008 [27]	Scotland	86% of sleep medicine specialists believed that dentists could contribute to the screening and referral of patients with OSA, as well as provide oral appliances.
Meenakshi et al., 2016 [29]	India	98% of medical practitioners lacked awareness of OAT for managing OSA. The referral rate to orthodontists for OSA management was 1.2%.
Alrejaye et al., 2022 [28]	Saudi Arabia	31% of medical practitioners lacked awareness of OAT for managing OSA. In total, 71.2% recognized the role of dentists in providing OAT.
This study	Thailand	20% of physicians did not recognize MADs as a treatment option for OSA patients, 36.7% regularly prescribed it, and 57.7% had specific dentists for referrals.

## Data Availability

The datasets generated and/or analyzed during the current study are available from the corresponding author upon reasonable request.

## Supplementary Materials

The following supporting information can be downloaded at https://www.mdpi.com/article/10.3390/dj13020063/s1. Table S1: Questionnaire used in this investigation.
