# Supplementary material for: A Survey of Physicians’ Knowledge and Practices Towards Oral Appliance Therapy for Obstructive Sleep Apnea Treatment
Source: Dent J (Basel). 2025 Jan 30;13(2):63. doi: 10.3390/dj13020063 (PMC11854440; doi:10.3390/dj13020063)
Supplement: Supplementary file 1 [file dentistry-13-00063-s001.zip › dentistry-3357522-supplementary.pdf]

Supplementary Material S1

Questionnaire

Part 1: Demographic data

No.	Questions
1.1	Sex<br>o Male<br>o Female
1.2	Age (years)<br>………………………………………………………………………………………
1.3	What year did you receive your Doctor of Medicine degree (M.D.)? (e.g. 2530)<br>………………………………………………………………………………………
1.4	What is your current professional status?<br>o Practicing specialist physician<br>o Practicing non-specialist physician<br>o Resident / Fellow
1.5	What is your specialty?<br>*(You may select more than one answer.)*<br>o General practitioner<br>o Sleep medicine<br>o Pediatrics<br>o Otolaryngology<br>o Psychiatry<br>o Neurology<br>o Pulmonology<br>o Internal medicine<br>o Family medicine<br>o Other, please specify ………………………………..................................
1.6	Where do you work?<br>*(You may select more than one answer.)*<br>o Government hospital or non-profit hospital<br>o Private hospital<br>o Private clinic<br>o Medical school<br>o Other, please specify ………………………………..................................

Part 2: Education and training experiences of oral appliances for OSA treatment.

No.	Questions
2.1	Are you a trained sleep medicine specialist?<br>o Yes<br>o No
2.2	Did you receive any education or attend any lecture/seminar/scientific meeting regarding oral appliances and orthodontic therapy for OSA treatment during your undergraduate medical program?<br>o Yes<br>o No
2.3	How did you receive any education or trainings in oral appliances and orthodontic therapy for OSA treatment?<br>*(You may select more than one answer.)*<br>o I have not received any education or trainings in oral appliances and orthodontic therapy for OSA treatment.<br>o Class/lecture/seminar in undergraduate medical education<br>o Class/lecture/seminar in postgraduate medical education<br>o Journals in medicine<br>o Journals in dentistry<br>o Academic conferences and meetings<br>o Social media<br>o Other, please specify …………………………….................................
2.4	Are you familiar with any of the following oral appliances?<br>*(You may select more than one answer.)*<br>o Mandibular advancement device (MAD)<br>o Tongue retaining device (TRD)<br>o Rapid maxillary expansion (RME)<br>o Orthodontic functional appliances (FA)

Part 3: Awareness and usage of various types of oral appliances for OSA treatment.

No.	Questions
3.1	To the best of your knowledge, which of the following is/ are used for OSA treatment in adult patients?<br>*(You may select more than one answer.)*<br>o Mandibular advancement device (MAD)<br>o Tongue-retaining device (TRD)<br>o Rapid maxillary expansion (RME)<br>o Orthodontic functional appliances (FA)<br>o None of these
3.2	To the best of your knowledge, which of the following is/are used for OSA treatment in children?<br>*(You may select more than one answer.)*<br>o Mandibular advancement device (MAD)<br>o Tongue-retaining device (TRD)<br>o Rapid maxillary expansion (RME)<br>o Orthodontic functional appliances (FA)<br>o None of these

Part 4: Indications and usage of oral appliances for OSA treatment.

No.	Questions
4.1	In adult patients with primary snoring, oral appliance therapy can be recommended as a first-line treatment.<br>o Strongly disagree<br>o Disagree<br>o Agree<br>o Strongly agree<br>o I do not know
4.2	In adult patients with mild to moderate OSA, oral appliance therapy can be recommended as a first-line treatment.<br>o Strongly disagree<br>o Disagree<br>o Agree<br>o Strongly agree<br>o I do not know
4.3	In adult patients with severe OSA and failed CPAP therapy, oral appliance therapy can be recommended.<br>o Strongly disagree<br>o Disagree<br>o Agree<br>o Strongly agree<br>o I do not know

Part 5: Perceived effectiveness of oral appliances for OSA treatment.

No.	Questions
5.1	In adult patients with OSA, oral appliance therapy improves the apnea hypopnea index (AHI)/respiratory disturbance index (RDI) and/or oxygen saturation.<br>o Strongly disagree<br>o Disagree<br>o Agree<br>o Strongly agree<br>o I do not know
5.2	In adult patients with OSA, oral appliance therapy is effective in improving health outcomes such as reducing blood pressure.<br>o Strongly disagree<br>o Disagree<br>o Agree<br>o Strongly agree<br>o I do not know
5.3	In adult patients with OSA, oral appliance therapy improves quality of life.<br>o Strongly disagree<br>o Disagree<br>o Agree<br>o Strongly agree<br>o I do not know
5.4	In adult patients with OSA, oral appliance therapy improves daytime sleepiness.<br>o Strongly disagree<br>o Disagree<br>o Agree<br>o Strongly agree<br>o I do not know
5.5	In adult patients with OSA, oral appliance users report better compliance and adherence compared to PAP users.<br>o Strongly disagree<br>o Disagree<br>o Agree<br>o Strongly agree<br>o I do not know

5.6	In children with OSA, rapid maxillary expansion (RME) improves the apnea hypopnea index (AHI) and reduces OSA severity.<br>o Strongly disagree<br>o Disagree<br>o Agree<br>o Strongly agree<br>o I do not know
5.7	In children with OSA, orthodontic functional appliances therapy improves the apnea hypopnea index (AHI) and reduces OSA severity.<br>o Strongly disagree<br>o Disagree<br>o Agree<br>o Strongly agree<br>o I do not know

Part 6: Referral practice of oral appliances for OSA treatment.

No.	Questions
6.1	Have you ever referred any patient with OSA for oral appliances and/or orthodontic therapy?<br>o Yes<br>o No<br>*(If the answer is 'Yes' please skip to question 4.3. If not, please answer next question.)*
6.2	What are reasons for not referring patients with OSA for oral appliances and orthodontic therapy?<br>*(You may select more than one answer.)*<br>o I do not see patient with OSA.<br>o I can treat patients with other treatment modality.<br>o I do not know about oral appliances and orthodontic therapy.<br>o I am not certain about the efficacy and benefits of oral appliances and orthodontic therapy.<br>o I do not know to whom and/or where I should refer my patients.<br>o There is no dentist that offer this type of treatment in my geographical area.<br>o Other, please specify ……………………………..................................<br>*(Please skip to part 5)*
6.3	Do you have dentist(s) that you regularly refer patients for treatment of OSA?<br>o Yes<br>o No
6.4	In the past 12 months, how many times have you referred adult patients with OSA for oral appliance therapy?<br>o Never<br>o Less than 5 times<br>o 5 to 10 times<br>o 11 to 20 times<br>o More than 20 times
6.5	In the past 12 months, how many times have you referred children with OSA for orthodontic therapy?<br>o Never<br>o Less than 5 times<br>o 5 to 10 times<br>o 11 to 20 times<br>o More than 20 times

Part 7: Decision making and treatment planning toward oral appliances for OSA treatment.

No.	Questions
7.1	In adult patients with OSA, what treatment options do you usually recommend? *(You may select more than one answer.)*<br>o Oral appliance therapy<br>o CPAP<br>o Behavioral modification<br>o Surgical treatment<br>o Other, please specify ………………………………..................................
7.2	What is/are your criteria when referring patients for oral appliance therapy? *(You may select more than one answer.)*<br>o Severity of OSA<br>o Patient preference<br>o CPAP intolerance<br>o Underlying diseases (e.g. cardiovascular diseases, neurodegenerative disease, epilepsy, etc.)<br>o Affordability<br>o Insurance coverage<br>o Patients have restriction to other treatment options.<br>o Other, please specify ………………………………..................................
7.3	Which of the following diagnosis that you consider referring adult patients with OSA for oral appliance therapy?<br>*(You may select more than one answer.)*<br>o I never refer adult patients with OSA for oral appliance therapy.<br>o Patient is diagnosed with primary snoring.<br>o Patient is diagnosed with mild OSA.<br>o Patient is diagnosed with moderate OSA<br>o Patient is diagnosed with severe OSA<br>o Other, please specify……………………………….................................
7.4	Which of the following diagnosis that you consider recommending oral appliance therapy as the first-line treatment for adult patients with OSA? *(You may select more than one answer.)*<br>o I never refer adult patients with OSA for oral appliance therapy as a first-line treatment.<br>o Patient is diagnosed with primary snoring.<br>o Patient is diagnosed with mild OSA.<br>o Patient is diagnosed with moderate OSA.<br>o Patient is diagnosed with severe OSA.<br>o Other, please specify ………………………………..................................

7.5	In children with mild OSA, what treatment options do you usually recommend? *(You may select more than one answer.)*<br>o Orthodontic therapy (e.g., RME, OFA, etc.)<br>o Adenotonsillectomy<br>o PAP therapy<br>o Medications<br>o Others, please specify …………………………….................................
7.6	In children with moderate to severe OSA, what treatment options do you usually recommend? *(You may select more than one answer.)*<br>o Orthodontic therapy (e.g., RME, OFA, etc.)<br>o Adenotonsillectomy<br>o PAP therapy<br>o Medications<br>o Others, please specify…………………………….................................
7.7	What is/are the information about oral appliance therapy that you usually explain to your patients before making a referral to a dentist?<br>*(You may select more than one answer.)*<br>o Cost<br>o Insurance coverage<br>o Time needed for the treatment<br>o Treatment mechanism<br>o Types of appliances<br>o Efficacy/effectiveness<br>o Advantages<br>o Disadvantages<br>o Complications<br>o Others, please specify …………………………….................................
7.8	In your opinion, who is involved in multidisciplinary team to manage patients with OSA? *(You may select more than one answer.)*<br>o Sleep medicine specialist<br>o Cardiologists<br>o Neurologist<br>o Pulmonologist<br>o Otolaryngologist<br>o Endocrinologist<br>o Bariatric surgeon<br>o Pediatrician<br>o Dental sleep medicine specialist<br>o Orthodontist<br>o Maxillofacial surgeon<br>o Dentist<br>o Others, please specify…………………………….................................

Part 8: Perceived importance of oral appliances and orthodontic therapy as an option for OSA treatment.

No.	Questions
8.1	Do you think that physicians should be educated in oral appliances and orthodontic therapy for OSA treatment? And why? (Please provide reason(s) for your answer.)<br>………..<br>………..<br>………..<br>………..
